# Sustained clinically meaningful efficacy and consistent safety in patients with agitation associated with dementia due to Alzheimer’s disease treated with brexpiprazole: a 24‐week *post hoc* analysis

**DOI:** 10.1002/alz70861_108748

**Published:** 2025-12-23

**Authors:** Anton P Porsteinsson, Sanjeda R Chumki, Anton M Palma, Malaak Brubaker, David Wang, Zhen Zhang, Pedro Such, C Brendan Montano

**Affiliations:** ^1^ Alzheimer’s Disease Care, Research and Education Program (AD‐CARE), Rochester, NY USA; ^2^ Otsuka Pharmaceutical Development & Commercialization Inc., Princeton, NJ USA; ^3^ Lundbeck LLC, Deerfield, IL USA; ^4^ H. Lundbeck A/S, Valby, Copenhagen Denmark; ^5^ Connecticut Clinical Research, Cromwell, CT USA

## Abstract

**Background:**

Agitation symptoms are common and burdensome in patients with Alzheimer’s dementia. In these vulnerable patients, it is important to sustain treatment effects over the longer term while maintaining a favorable safety profile. This *post hoc* analysis aimed to evaluate clinically meaningful efficacy, and safety, of brexpiprazole throughout 24 weeks of treatment.

**Method:**

Data were included from a Phase 3, 12‐week, randomized, placebo‐controlled trial of brexpiprazole 2 or 3 mg/day in patients with agitation associated with dementia due to Alzheimer’s disease (ClinicalTrials.gov: NCT03548584 [Trial 213]) and a 12‐week extension trial (NCT03594123 [Trial 182]). Trial 182 enrolled patients who completed treatment with brexpiprazole or placebo in Trial 213; thereafter, patients received brexpiprazole 2 or 3 mg/day. In this *post hoc* analysis, the proportion of patients who achieved a sustained clinically meaningful response (20‐point reduction in Cohen‐Mansfield Agitation Inventory Total score maintained to trial end) was evaluated over 12 weeks (brexpiprazole versus placebo) and 24 weeks (‘prior brexpiprazole’ [received brexpiprazole in both trials] versus ‘prior placebo’ [received placebo in Trial 213 and brexpiprazole in Trial 182]). Safety was assessed in the prior brexpiprazole (24‐week brexpiprazole) subgroup only, reflecting the longest evaluable brexpiprazole treatment duration. The incidence of treatment‐emergent adverse events (TEAEs), and median TEAE duration, were assessed. Analyses of time to first occurrence of any TEAE were conducted using descriptive statistics and Kaplan–Meier methodology.

**Result:**

A sustained clinically meaningful response was achieved by 49.3% (brexpiprazole; n=225) versus 32.8% (placebo; n=116) at Week 12, and 75.5% (prior brexpiprazole; n=159) versus 68.4% (prior placebo; n=95) at Week 24. In the prior brexpiprazole subgroup (*n* =163), 48.5% reported TEAEs over 24 weeks. Median (interquartile range) TEAE duration was 3 (1–8) days and time‐to‐first event was 7.4 (3–12) weeks. Kaplan–Meier curves indicated that, among patients who had not already experienced a TEAE in the randomized trial, TEAEs were rare throughout the extension trial.

**Conclusion:**

Brexpiprazole 2 or 3 mg/day was associated with sustained clinically meaningful efficacy over 24 weeks in patients with agitation associated with dementia due to Alzheimer’s disease. Longer‐term treatment with brexpiprazole over the 24‐week period was not associated with additional safety signals.

